# Low endogenous testosterone levels are associated with the extend of lymphnodal invasion at radical prostatectomy and extended pelvic lymph node dissection

**DOI:** 10.1007/s11255-021-02938-z

**Published:** 2021-07-06

**Authors:** Antonio Benito Porcaro, Clara Cerrato, Alessandro Tafuri, Alberto Bianchi, Sebastian Gallina, Rossella Orlando, Nelia Amigoni, Riccardo Rizzetto, Alessandra Gozzo, Filippo Migliorini, Stefano Zecchini Antoniolli, Carmelo Monaco, Matteo Brunelli, Maria Angela Cerruto, Alessandro Antonelli

**Affiliations:** 1 Department of Urology, Ospedale Civile Maggiore, Polo Chirurgico Confortini, University of Verona, Azienda Ospedaliera Universitaria Integrata Verona, Piazzale Stefani 1, 37126 Verona, Italy; 2 Department of Neuroscience, Imaging and Clinical Sciences, University G. D’Annunzio of Chieti-Pescara, Chieti, Italy; 3 Department of Pathology, University of Verona, Azienda Ospedaliera Universitaria Integrata Verona, Piazzale Stefani 1, 37126 Verona, Italy

**Keywords:** Prostate cancer, Radical prostatectomy, Extended lymph node dissection, Endogenous testosterone, Locally advanced prostate cancer, Lymph node metastases

## Abstract

**Objective:**

To investigate clinical factors associated to lymphnodal metastasis load in patients who underwent to radical prostatectomy (RP) and extended pelvic lymph node dissection (ePLND).

**Materials and methods:**

Between November 2014 and December 2019, ET was measured in 617 consecutive patients not under androgen deprivation therapy who underwent RP and ePLND. Lymphnode invasion (LNI) was codified as not present (*N* = 0) or with one (*N* = 1) or more than one metastatic node (*N* > 1). The risk of multiple pelvic lymph node metastasis (*N* > 1, mPLNM) was assessed by comparing it to the other two groups (*N* > 1 vs. *N* = 0 and *N* > 1 vs. *N* = 1). Then, we assessed the association between ET and lymphnode invasion for standard predictors, such as PSA, percentage of biopsy positive cores (BPC), tumor stage greater than 1 (cT > 1) and tumor grade group greater than two (ISUP > 2).

**Results:**

Overall, LNI was detected in 70 patients (11.3%) of whom 39 (6.3%) with *N* = 1 and 31 (5%) with *N* > 1. On multivariate analysis, ET was inversely associated with the risk of *N* > 1 when compared to both *N* = 0 (odds ratio, OR 0.997; CI 0.994–1; *p* = 0.027) as well as with *N* = 1 cases (OR 0.994; 95% CI 0.989–1.000; *p* = 0.015).

**Conclusions:**

In clinical PCa, the risk of mPLNM was increased by low ET levels. As ET decreased, patients had an increased likelihood of mPLNM. Because of the inverse association between ET and mPLNM, higher ET levels were protective against aggressive disease. The influence of locally advanced PCa with high metastatic load on ET levels needs to be explored by controlled trials.

## Introduction

Prostate cancer (PCa) worldwide is the second most incident solid cancer and represents a pivotal health related problem for the aging male [1]. According to guidelines, surgery represents a valid therapeutic option for most of non-metastatic PCa patients with a life-expectancy over 10–15 years. When surgery is selected, lymphnode dissection with an extended template (ePLND) should be performed when the risk of lymphnodal invasion exceeds 5%, according to the estimation provided by integrated predictive systems based on pre-operative features [2]. Any available diagnostic tool to date suffers from significant inaccuracy concerning lymphnodal staging in PCa and, therefore, the main objective of ePLND remains the correct definition of lymphnodal involvement [3]. Nonetheless, it also provides some additional prognostic information by estimating the burden of lymphnodal involvement and, probably, could exert a curative role in case of very limited (< 2) involvement [4].

Endogenous testosterone (ET) levels have a well-established role with the development and progression of PCa, showing significant associations with tumor biology. The relationship between pre-operative testosterone serum levels and prostate cancer stage and grade is debated. Indeed, some evidence suggests a linear correlation between pre-operative androgen levels and aggressiveness of PCA [5, 6]. However, other authors found that low levels of testosterone are associated with more aggressive PCA [7–10]. Specifically, in the recent literature, a very few studies are present on the relationship between testosterone levels and nodal invasion.

With the present study we investigated the hypothesis that ET levels could be associated with the lymphnodal metastatic burden in patients undergoing RP with ePLND.

## Materials and methods

### Patients and methods

The study was retrospective and approved by internal Institutional Review Board. Informed signed consent was obtained by patients. Data were collected prospectively but evaluated retrospectively. In a period ranging from November 2014 to December 2019, all consecutive patients received ET measure before RP within a prospective project that aimed at collecting this data for all surgical candidates. Overall, 617 consecutive patients undergone RP plus ePLND were considered for the present analysis.

ET (ng/dL) was measured at our lab at least one month after biopsies between 8.00 and 8.30 a.m. by radioimmunoassay.

PSA (ng/mL), age (years), body mass index (BMI; kg/m^2^), prostate volume (PV, mL) and biopsy positive cores (BPC; percentage) were also calculated in each case. Clinically and pathologically, tumors were staged according to the TNM system [2, 4]. Procedures were performed by the robot assisted approach (RARP) in 534 out of 617 patients (86.5%). ePLND was performed according to guideline recommendations [2, 4]. The decision to perform an extended lymph node dissection was mainly based on pre-operative nomograms showing a risk of lymph node invasion greater than 5% [11]. In the low and intermediate risk categories, ePLND was decided according to EAU recommendations and factors predicting tumor upgrading [12–14]. Lymph node dissection was developed according to a standard anatomical template including external iliac, obturator, Cloquet’s and Marcille’s regions [15, 16]. Specimens were evaluated for tumor grade and stage, surgical margins, number of removed and metastatic lymph nodes [2, 4]. Tumors were graded according to the International Society of Urological Pathology (ISUP) system; furthermore, tumor load (TL) was also evaluated as percentage of cancer involving the prostate gland.

Perioperative surgical risk was evaluated by the American Society of Anesthesiologists (ASA) score system [17]. Postoperative surgical complications were graded according to the Clavien–Dindo system for a period of 90 days [18]. Hospital readmission events were also evaluated.

### Study design and statistical methods

The study investigated on association between ET levels and aggressive PCA, which was defined by the event of detecting multiple pelvic lymph node metastases (mPLNM) after ePLND.

As such, pathological status of dissected lymph nodes was codified at three levels including no LNI (*N* = 0), one lymph node metastasis (*N* = 1) and more than one metastatic lymph node (*N* > 1). According to their distributions, continuous variables were represented as medians with relative interquartile ranges (IQR) while categorical factors were assessed as frequencies (percentages). Associations of ET with the risk of pelvic lymph node metastases (overall, single and multiple) were assessed by the binomial and multinomial logistic regression model (univariate and multivariate analysis). Receiver operating characteristics (ROC) curves with relative areas under the curve (AUC) were evaluated for independent variables predicting the risk of mPLNM. Accuracy of multivariate models predicting the risk of mPLNM were assessed by classifications after stratifying the exposure variable (ET) into quartile levels and after adjusting for independent standard clinical predictors. The software used to run the analysis was IBM-SPSS version 26. All tests were two-sided with *p* < 0.05 considered to indicate statistical significance.

## Results

### a) Demographics of overall, pN0 vs pN + PCa population

Demographics of overall and stratified patient population is reported in Table 1. Clinical classes were low risk in 78 patients (12.6%), intermediate risk in 343 subjects (55.6%) and high risk in 196 cases (31.8%). ASA system scored one in 58 cases (9.4%), two in 506 patients (82%) and three in 53 subjects (8.6%). Clavien–Dindo complications were grade 1–2 in 152 patients (24.6%) and grade 3 up to 4a in 42 cases (6.8%). Median (IQR) of length of hospital stay was 4 days (4–5 days). Readmissions occurred in 5.1% of cases. Overall, LNI was detected in 70 patients (11.3%) of whom 39 (6.3%) with one lymph node metastasis and 31 (5%) having more than one metastatic lymph node.

**Table 1:** Baseline patient characteristics by levels of lymph node invasion

	Total patients	Number of metastases in dissected loco-regional lymph nodes			
		Zero	One or more than one	One	More than one
*N* (%)	617	547 (88.7)	70 (11.3)	39 (6.3)	31 (5)
Clinical factors (*)					
Endogenous testosterone; ET (ng/dL)	432.4 (339.8–535.3)	433 (340.3–538.9)	416.3 (332.5–493.5)	443.8 (363.1–536.6)	382.2 (330.6–465)
Body mass index; BMI (kg/m^2^)	25.6 (23.7–28.1)	25.6 (23.7–28)	25.9 (24.3–28.3)	25 (23.9–27)	27.4 (24.7–29.9)
Age (years)	66 (61.70)	66 (61–70)	67 (62–71)	68 (63–71)	66 (61–71)
Prostate specific antigen; PSA (ng/mL)	6.7 (4.9–9.4)	6.5 (4.9–8.9)	8.4 (5–14.3)	7.2 (5–11.2)	12.1 (6.8–20.5)
Prostate volume; PV (mL)	40 (30–54)	40 (30–53)	45 (32.8–59.3)	45 (30–60)	45 (38–59)
Biopsy positive cores; BPC (%)	33 (21–50)	32.1 (21–50)	50 (32.5–73.5)	45 (25–71)	58 (40–80)
International Society of Urologic Pathology (ISUP) tumor grade system ISUP < 3	377 (64.1)	349 (63.8)	28 (40)	19 (48.7)	9 (29)
ISUP > 2	240 (38.9)	198 (36.2)	42 (60)	20 (51.3)	22 (71)
Tumor stage (cT)					
cT1	331 (53.6)	310 (56.7)	21 (30)	11 (28.2)	10 (32.3)
cT2	286 (46.4)	237 (43.2)	49 (70)	28 (71.8)	21 (67.7)
Nodal stage (cN)					
cN0	583 (94.5)	518 (94.7)	65 (92.9)	38 (97.4)	27 (87.1)
cN1	34 (5.5)	29 (5.3)	5 (7.1)	1 (2.6)	4 (12.9)
Pathological factors (*)					
Prostate weight; PW (grams)	52 (42–65)	51 (41–65)	55.3 (45–71.3)	55 (45–70.8)	55.6 (45–74)
Tumor load; TL (%)	20 (10–30)	20 (10–30)	30 (20–50)	30 (20–50)	40 (20–50)
International Society of Urologic Pathology (ISUP) tumor grade system ISUP < 3	442 (71.6)	232 (42.4)	3 (4.3)	2 (5.1)	1 (3.2)
ISUP > 2	175 (28.4)	315 (57.6)	67 (95.7)	37 (94.9)	30 (96.8)
Pathological tumor stage (pT)					
pT2	453 (73.5)	426 (77.9)	27 (38.6)	18 (46.2)	9 (29)
pT3a	70 (11.3)	63 (11.5)	7 (10)	5 (12.8)	2 (6.5)
pT3b	94 (15.2)	58 (10.6)	36 (51.4)	16 (41)	20 (64.5)
Positive surgical margins (PSM) no PSM	442 (71.6)	409 (74.8)	33 (47.1)	17 (43.6)	16 (51.6)
PSM	175 (28.4)	138 (25.2)	37 (52.9)	22 (56.4)	15 (48.4)
Number of dissected lymph nodes; LN (*n*)	24 (18–32)	24 (17.7–31)	28 (22–36)	27 (21–36)	31 (24–37)

(*), Distribution of continuous factors are reported as medians with relative interquartile (IQR) ranges while categorical factors are reported as frequency with relative percentage (%)

### b) Inverse association between ET and risk of mPLNM (univariate analysis)

Associations between factors and risk of LNI are reported in Table 2. As shown, standard factors confirmed their association with the risk of pN1 disease. When patients were stratified according to levels of metastatic load, ET and BMI associated with the risk of mPLNM when compared to pN0 cases; furthermore, the association was inverse for the former (odds ratio, OR 0.997; 95% CI 0.994–1.000; *p* = 0.029) and direct for the latter (OR 1.134; 95% CI 1.023–1.256; *p* = 0.017). In metastatic patients, the risk of mPLNM was only predicted by ET, BMI and BPC, as well. Considering factors related to the surgical specimen, high tumor grade (ISUP > 2), seminal vesicle invasion and positive surgical margins associated with features of LNI. Furthermore, the number of dissected lymph nodes were higher for cases with mPLNM compared to negative cases; however, the significance disappeared when comparing between metastatic cases.

**Table 2:** Associations of clinical and pathological factors with the risk of lymph node metastases in patients with clinical prostate cancer (univariate analysis)

	Metastases in one or more lymph nodes vs none		Metastases in one lymph node vs none		Metastases in more than one lymph node vs none		Metastases in more than one lymph node vs one	
Clinical factors	OR (95% CI)	*p *value	OR (95% CI)	*p *value	OR (95% CI)	*p *value	OR (95% CI)	*p *value
ET	0.999 (0.997–1.001)	0.209	1.000 (0.998–1.002)	0.864	0.997 (0.994–1.000)	0.029	0.996 (0.992–1.000)	0.053
BMI	1.032 (0.957–1.112)	0.418	0.947 (0.854–1.050)	0.301	1.134 (1.023–1.256)	0.017	1.213 (1.035–1.418)	0.017
Age	1.025 (0.985–1.067)	0.223	1.040 (0.985–1.097)	0.154	1.008 (0.952–1.067)	0.781	0.969 (0.911–1.015)	0.154
PSA	1.053 (1.025–1.082)	< 0.0001	1.030 (0.989–1.072)	0.156	1.072 (1.038–1.107)	< 0.0001	1.041 (0.997–1.088)	0.069
PV	1.010 (0.998–1.022)	0.118	1.007 (0.990–1.023)	0.426	1.013 (0.997–1.030)	0.12	1.007 (0.984–1.029)	0.561
BPC	1.027 (1.017–1.037)	< 0.0001	1.019 (1.006–1.032)	0.005	1.038 (1.023–1.052)	< 0.0001	3.565 (1.321–9.610)	0.012
ISUP < 3	Ref							
ISUP > 2	2.644 (1.589–4.318)	< 0.0001	1.855 (0.967–3.560)	0.063	4.309 (1.946–9.540)	< 0.0001	2.322 (0.856–6.299)	0.098
cT < 2	Ref							
cT > 1	3.052 (1.781–5.229)	< 0.0001	3.329 (1.624 -6.824)	0.001	2.747 (1.270–5.943)	0.01	0.825 (0.296–2.303)	0.713
cN0	Ref							
cN1	1.374 (0.514–3.674)	0.527	0.470 (0.062–3.545)	0.464	2.646 (0.868–8.067)	0.087	5.630 (0.596–53.207)	0.132
Pathological factors	OR (95% CI)	*p *value	OR (95% CI)	*p *value	OR (95% CI)	*p *value	OR (95% CI)	*p *value
PW	1.011 (1.000–1.023)	0.055	1.012 (0.995–1.025)	0.191	1.013 (0.997–1.029)	0.124	1.003 (0.982–1.024)	0.804
TL	1.040 (1.027–1.053)	< 0.0001	1.039 (1.023–1.054)	< 0.0001	1.042 (1.024–1.058)	< 0.00001	1.002 (0.983–1.022)	0.813
ISUP < 3	Ref							
ISUP > 2	16.449 (5.110–52.945)	< 0.0001	13.625 (3.251–57.104)	< 0.0001	22.095 (2.992–163.192)	0.002	1.622 (0.140–18.730)	0.699
pT2	Ref							
pT3a	1.735 (0.733–4.194)	0.207	1.878 (0.674–5.288)	0.228	1.503 (0.317–7.114)	0.608	0.800 (0.129–4.960)	0.811
pT3b	9.793 (5.541–17.307)	< 0.0001	6.529 (3.155–13.509)	< 0.0001	16.322 (7.095–37.550)	< 0.00001	2.500 (0.888–7.042)	0.083
NSM	Ref							
PSM	3.323 (2.001–5.519)	< 0.0001	3.835 (1.979–7.433)	< 0.0001	2.799 (1.339–5.678)	0.006	0.724 (0.281–1.868)	0.505
LN	1.031 (1.011–1.051)	0.002	1.022 (0.996–1.048)	0.098	1.041 (1.014–1.069)	0.003	1.019 (0.984–1.055)	0.292

### c) Low ET levels increased the risk of mPLNM (univariate analysis)

**Fig. 1:**
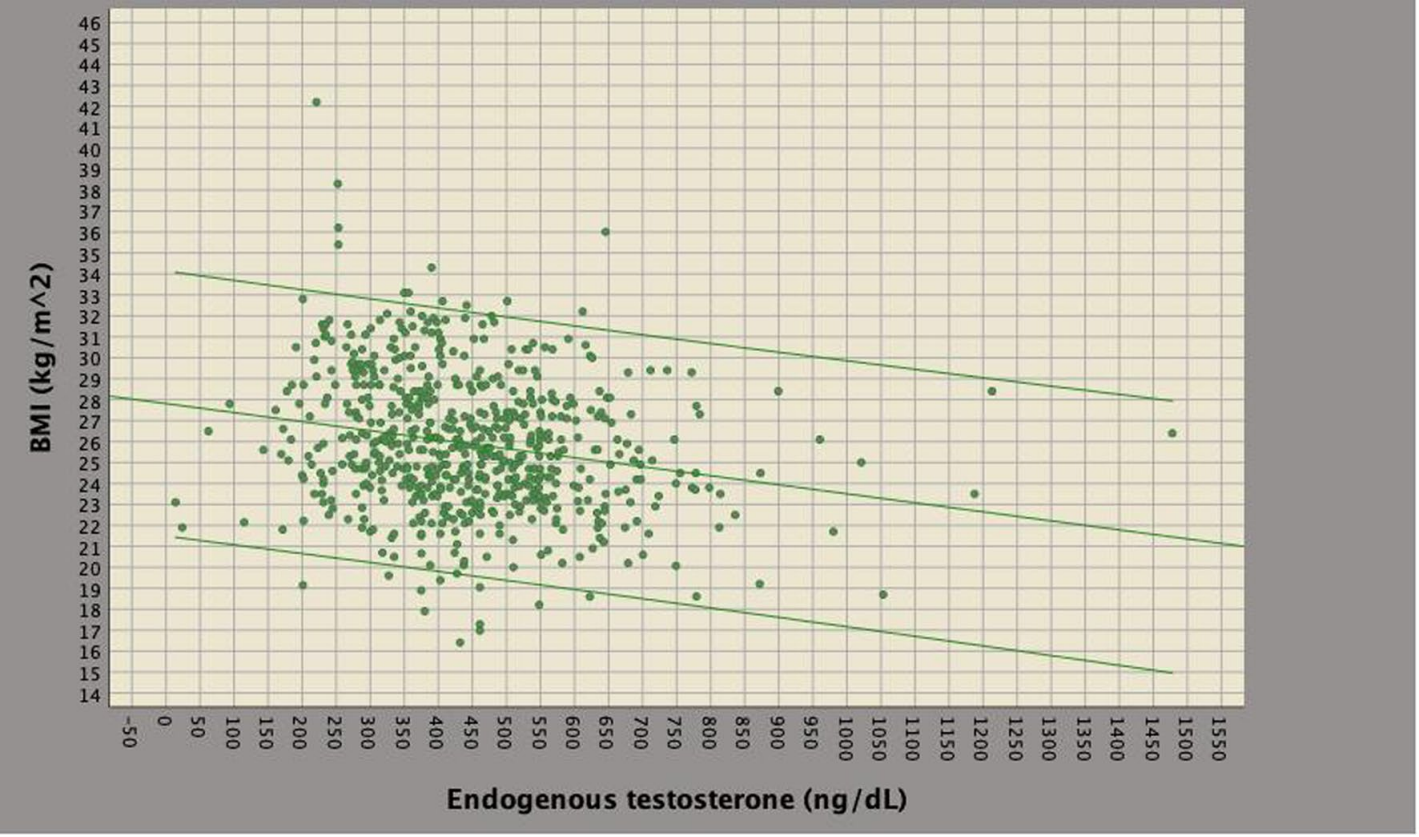
Scatterplot of endogenous testosterone (ET; ng/dL) versus body mass index (BMI; kg/m^2^) in 617 prostate cancer patients who underwent radical prostatectomy with extended pelvic lymph dissection. ET and BMI were inversely correlated (Pearson’s correlation coefficient, *r* =− 0.210; *p* < 0.0001). The regression lines with 95% confidence intervals are also reported

**Fig. 2:**
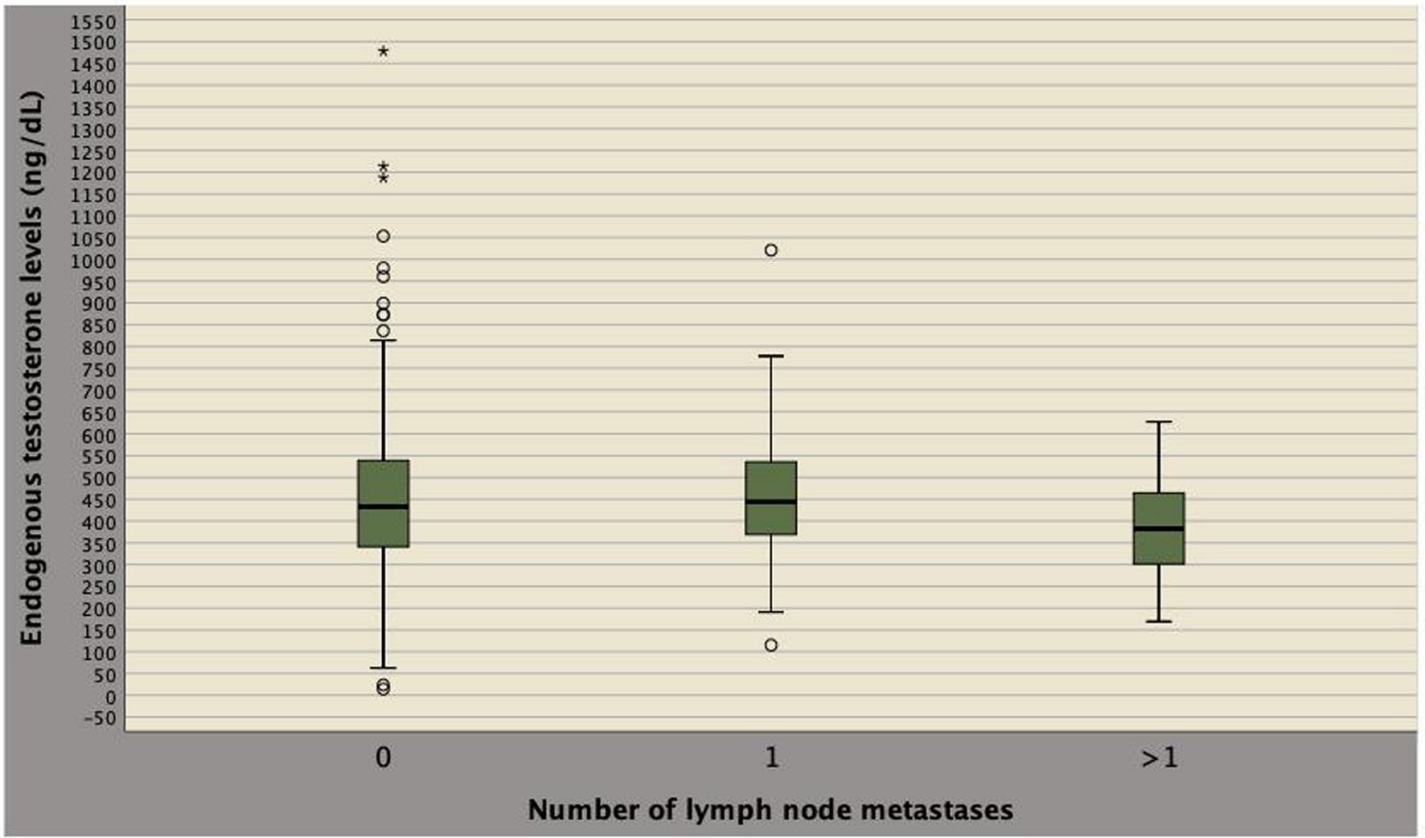
Box plots of endogenous testosterone (ET) levels stratified by the number of metastatic nodes. As shown, lower median ET levels were detected in patients with mPLNM when compared to both patients without or with only one lymph node invasion

**Fig. 3:**
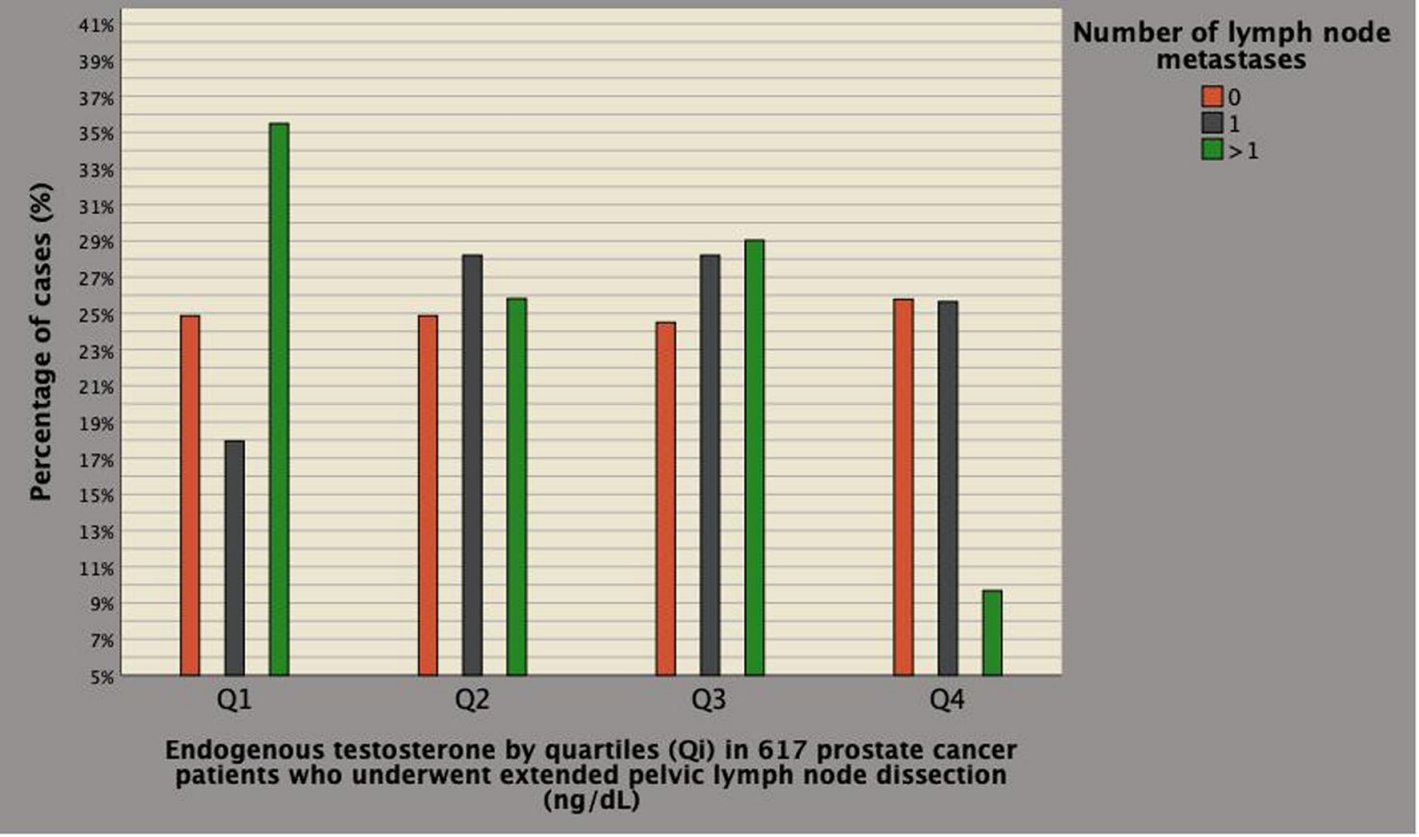
The figure shows the distributions of patients with multiple pelvic lymph metastases stratified endogenous testosterone (ET) quartiles; as illustrated, rates were higher in patients with ET levels within the first quartile (35.5%) compared to cases above the third quartile (9.7%). See also Table 3

As shown in Fig. 1, ET and BMI inversely correlated to each other. So far, ET was stratified by quartiles and associations were investigated on clinical and pathological factors (univariate analysis). As shown in Table 3, low ET levels associated with the risk of multiple LNI. Specifically, the risk of mPLNM increased by ET levels within the first quartile, compared to levels above the third quartile, for both cases with no LNI (OR 3.801; 95% CI 1.038–13.923; *p* = 0.044) as well subjects with one lymph node metastasis (OR 5.238; 95% CI 1.057–25.966; *p* = 0.043). Figure 2 illustrates box plots of ET levels stratified by the number of metastatic nodes. As shown, lower median ET levels were detected in patients with mPLNM when compared to patients without or with only one LNI. Figure 3 shows the distributions of mPLNM by ET quartiles; as illustrated, rates of multiple LNI were higher in patients with ET levels within the first quartile (35.5%) compared to cases with ET levels above the third quartile (9.7%).

**Table 3:** Associations of factors with endogenous testosterone levels stratified by quartiles in 617 patients who underwent extended pelvic lymph node dissection

	Endogenous testosterone by quartiles (ng/dL)			
	Q1: ≤ 340.1	Q2: 340.2–432	Q3: 433–535.2	Q4: > 535.3
*N* (%)	154 (25)	155 (25,1)	154 (25)	154 (25)
ET (ng/dL)	279.4 (231–307.5)	386.5 (364.5–505.4)	479.8 (459.5–505.4)	610.7 (558.8–683.9)
Clinical factors				
BMI (kg/m^2^)	26.4 (24.6–29.4)	26 (23.8–28.4)	25.3 (23.7–27.2)	24.7 (23.1–27.2)
OR (95% CI)	1.211 (1.125–1.304)	1.126 (1.047–1.272)	1.054 (0.980–1.134)	Ref
* p* value	< 0.0001	0.001	0.158	
Age (years)	67 (62–71)	65 (60–69.2)	65.5 (60–70)	67 (61–71)
OR (95% CI)	1.002 (0.967–1.038)	0.959 (0.926–0.993)	0.979 (0.945–1.014)	Ref
* p* value	0.92	0.02	0.237	
PSA (ng/mL)	6.8 (4.7–8.5)	6.8 (4.9–9.7)	7.2 (5.0–10.2)	6.2 (5.0–8.7)
OR (95% CI)	1.002 (0.978–1.027)	0.998 (0.972–1.024)	1.004 (0.980–1.028)	Ref
* p* value	0.859	0.871	0.751	
PV (mL)	40.5 (30–51.5)	38.1 (30–52)	40.1 (32.6–55)	41 (30–55)
OR (95% CI)	0.996 (0.984–1.018)	0.995 (0.983–1.007)	1.005 (0.993–1.017)	Ref
* p* value	0.545	0.445	0.399	
BPC (%)	37 (21–54.5)	33 (21–50)	33.3 (25–56.3)	31.1 (20–50)
OR (95% CI)	1.003 (0.993–1.013)	1.001 (0.991–1.011)	1.005 (0.995–1.014)	Ref
* p* value	0.55	0.863	0.341	
ISUP < 3 (ref)	93 (60.4)	101 (65.2)	98 (63.6)	85 (55.2)
ISUP > 2	61 (39.6)	54 (34.8)	56 (36.4)	69 (44.8)
OR (95% CI)	0.808 (0.514–1.271)	0.659 (0.416–1.042)	0.704 (0.446–1.112)	Ref
* p* value	0.356	0.074	0.132	
cT1 (ref)	74 (48.1)	84 (54.2)	86 (55.8)	87 (56.5)
cT > 1	80 (51.9)	71 (45.8)	68 (44.2)	67 (46.3)
OR (95% CI)	1.404 (0.896–2.199)	1.098 (0.701–1.719)	1.027 (0.655–1.610)	Ref
* p* value	0.139	0.684	0.909	
cN0 (ref)	143 (92.9)	148 (95.5)	149 (96.8)	143 (92.9)
cN1	11 (7.1)	7 (4.5)	5 (3.2)	11 (7.1)
OR (95% CI)	1.000 (0.420–2.380)	0.615 (0.232–1.630)	0.436 (0.448–1.287)	Ref
* p* value	0.999	0.328	0.133	
Pathological factors				
PW (grams)	52.3 (41–65)	50 (41–64)	50.6 (41.9–66.3)	55 (44.5–69)
OR (95% CI)	0.998 (0.986–1.009)	0.996 (0.984–1.007)	0.998 (0.987–1.009)	Ref
* p* value	0.668	0.448	0.998	
TL (%)	20 (10–30)	20 (10–30)	20 (10–30)	20 (10–30)
OR (95% CI)	0.996 (0.984–1.009)	0.995 (0.982–1.008)	0.995 (0.982–1.008)	Ref
* p* value	0.57	0.878	0.995	
ISUP < 3 (ref)	60 (39)	62 (39.7)	56 (36.4)	57 (37)
ISUP > 2	94 (61)	93 (60.3)	98 (63.6)	97 (63)
OR (95% CI)	0.921 (0.581–1.459)	0.881 (0.557–1.394)	1.028 (0.647–1.635)	Ref
* p* value	0.725	0.59	0.906	
pT2 (ref)	107 (69.5)	119 (76.8)	109 (70.8)	118 (76.6)
pT3a	23 (14.9)	16 (10.3)	18 (11.7)	13 (8.4)
OR (95% CI)	1.951 (0.942–4.043)	1.220 (0.562–2.469)	1.499 (0.701–3.203)	Ref
* p* value	0.072	0.614	0.296	
pT3b	24 (15.6)	20 (12.9)	27 (17.5)	23 (14.9)
OR (95% CI)	1.151 (0.614–2.158)	0.862 (0.450–1.654)	1.271 (0.688–2.348)	Ref
* p* value	0.662	0.656	0.444	
No PSM (ref)	115 (74.7)	106 (68.4)	118 (76.6)	103 (66.9)
PSM	39 (25.3)	49 (31.6)	36 (23.4)	51 (33.1)
OR (95% CI)	0.685 (0.418–1.123)	0.934 (0.580–1.504)	0.616 (0.373–1.018)	Ref
* p* value	0.134	0.778	0.059	
LN (*n*)	24 (18–31.3)	25 (18–32)	25 (18–32)	24 (17–32)
OR (95% CI)	1.003 (0.985 -1.022)	1.012 (0.994–1.030)	1.007 (0.989–1.026)	Ref
* p* value	0.73	0.203	0.45	
Number of lymph node metastases (*N*)				
*N* = 0 (ref)	136 (24.9)	136 (24.9)	134 (24.5)	141 (25.8)
*N* = 1	7 (17.9)	11 (28.2)	11 (28.2)	10 (25.6)
	0.726 (0.269–1.961)	1.140 (0.469–2.772)	1.157 (0.476–2.814)	Ref
	0.527	0.084	0.747	
*N* > 1	11 (35.5)	8 (25.8)	9 (29)	3 (9.7)
	3.801 (1.038–13.923)	2.765 (0.718–10.641)	3.157 (0.837–11.910)	Ref
	0.044	0.139	0.69	
*N* = 1 (ref)	7 (17.9)	11 (28.2)	11 (28.2)	10 (25.6)
*N* > 1	11 (35.5)	8 (25.8)	9 (29)	3 (9.7)
	5.238 (1.057–25.966)	2.424 (0.500–11.761)	2.727 (0.522–13.008)	Ref
	0.043	0.272	0.208	

Distribution of factors are reported as medians with relative interquartile (IQR) ranges while categorical factors are reported as frequency with relative percentages (%); see also Table 1 for legend of factors

*OR* odds ratio; *CI* confidence interval

### d) ET as an independent protective factor against the risk of mPLNM

On multivariate analysis, standard clinical parameters predicted overall LNI, as illustrated in Table 4. Compared to pN0 patients, patients with one metastasis were predicted only by BPC and cT > 1 while mPLNM, beyond standard factors, were predicted by ET with BMI losing significance.

**Table 4:** Associations of clinical and pathological factors with the risk of different levels of lymph node metastases in patients with clinical prostate cancer (multivariate analysis)

	Metastases in one or more lymph nodes vs none		Metastases in one lymph node vs none		Metastases in more than one lymph node vs none		Metastases in more than one lymph node vs one	
Clinical model	OR (95% CI)	*p *value	OR (95%CI)	*p *value	OR (95% CI)	*p *value	OR (95% CI)	*p *value
ET					0.997 (0.994–1.000)	0.027	0.994 (0.989–1.000)	0.015
BMI							1.257 (1.046–1.511)	0.015
Age								
PSA	1.039 (1.008–1.071)	0.013			1.048 (1.014–1.084)	0.005		
PV								
BPC	1.019 (1.008–1.030)	0.001	1.016 (1.003–1.030)	0.014	1.026 (1.010–1.043)	0.002		
ISUP < 3	Ref				Ref			
ISUP > 2	2.005 (1.166–3.447)	0.012			3.340 (1.412–7.804)	0.006		
cT < 2	Ref							
cT > 1	2.592 (1.480–4.540)	0.001	3.112 (1.511–6.409)	0.002				
cN0	Ref							
cN1								
Pathological model	OR (95%CI)	*p *value	OR (95% CI)	*p* value	OR (95% CI)	*p *value	OR (95% CI)	*p* value
PW								
TL	1.019 (1.004–1.035)	0.015	1.021 (1.003–1.040)	0.024				
ISUP < 3	Ref							
ISUP > 2	10.400 (3.085–35.062)	< 0.0001	9.401 (2.152–41.062)	0.003	12.246 (1,170–114,672)	0.013		
pT2	Ref							
pT3a								
pT3b	3.788 (2.079–6.902)	< 0.0001	2.508 (1.171–5.371)	0.018	8.513 (3.701–19.583)	< 0.0001		
No PSM	Ref							
PSM	1.817 (0.997–3.310)	0.051						
LN	1.038 (1.013–1.064	0.003	2.194 (1.047–4.598)	0.037	1.051 (1.015–1.089)	0.005		

*OR* odds ratio; *CI* confidence interval; see also Table 1 for legend of factors

**Fig. 4:**
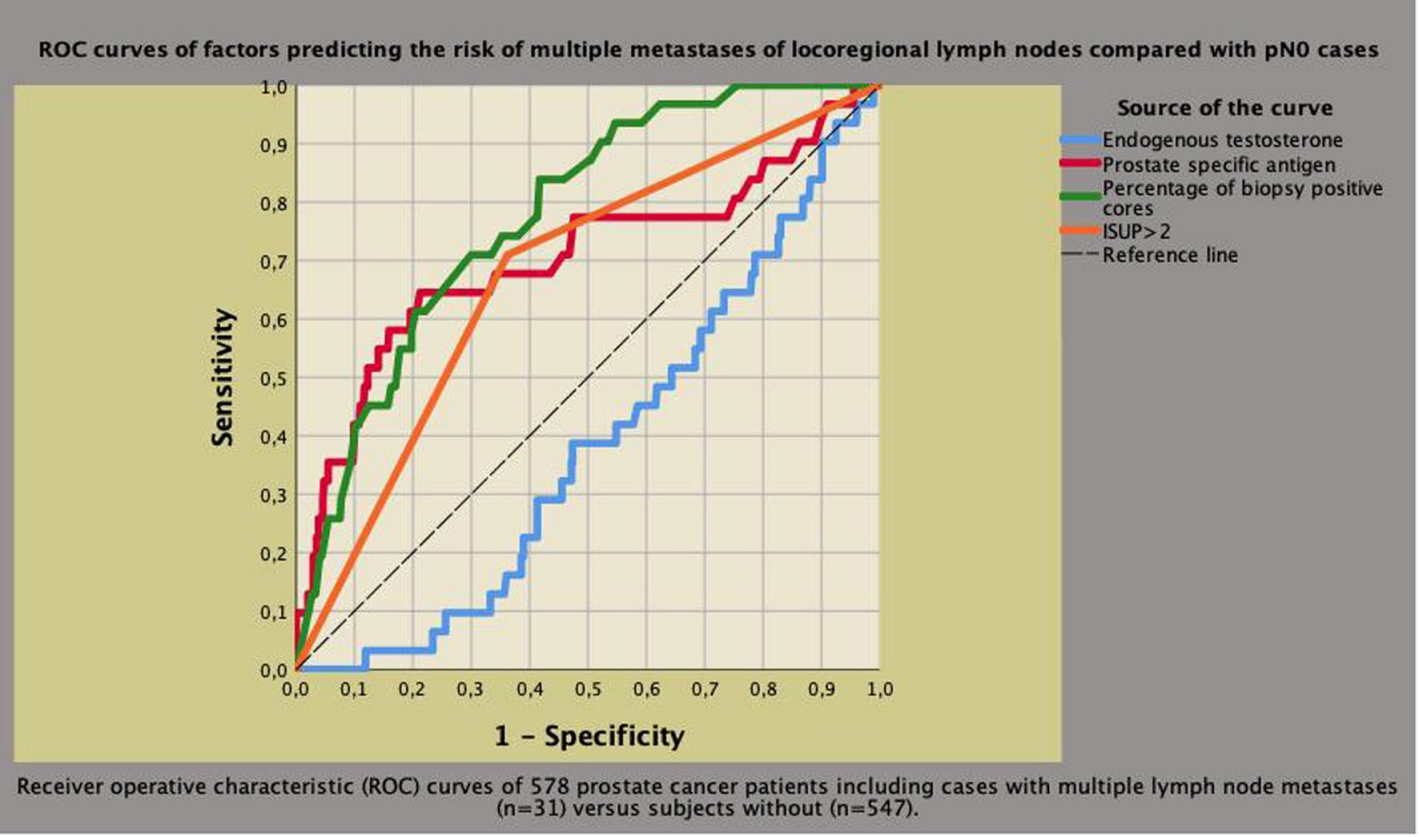
Receiver operating characteristic (ROC) curves of clinical factors predicting the risk of multiple pelvic lymph node metastases (mPLNM) compared to cases without any. Area under the curve (AUC) was 0.703 (95% CI 0.588–0.819; *p* = 0.001) for prostate specific antigen (PSA), 0.777 (95% CI 0.704–0.850; *p* < 0.0001) for biopsy positive cores (BPC), 0.674 (95% CI 0.578–0.769; *p* < 0.0001) for tumor grade group > 2 according to International Society of Urologic Pathology (ISUP) system and 0.382 (0.294–0.470; *p* = 0,027) for endogenous testosterone (ET). So far, higher ET levels were protective for the risk of mPLNM. See results section for further details

**Fig. 5:**
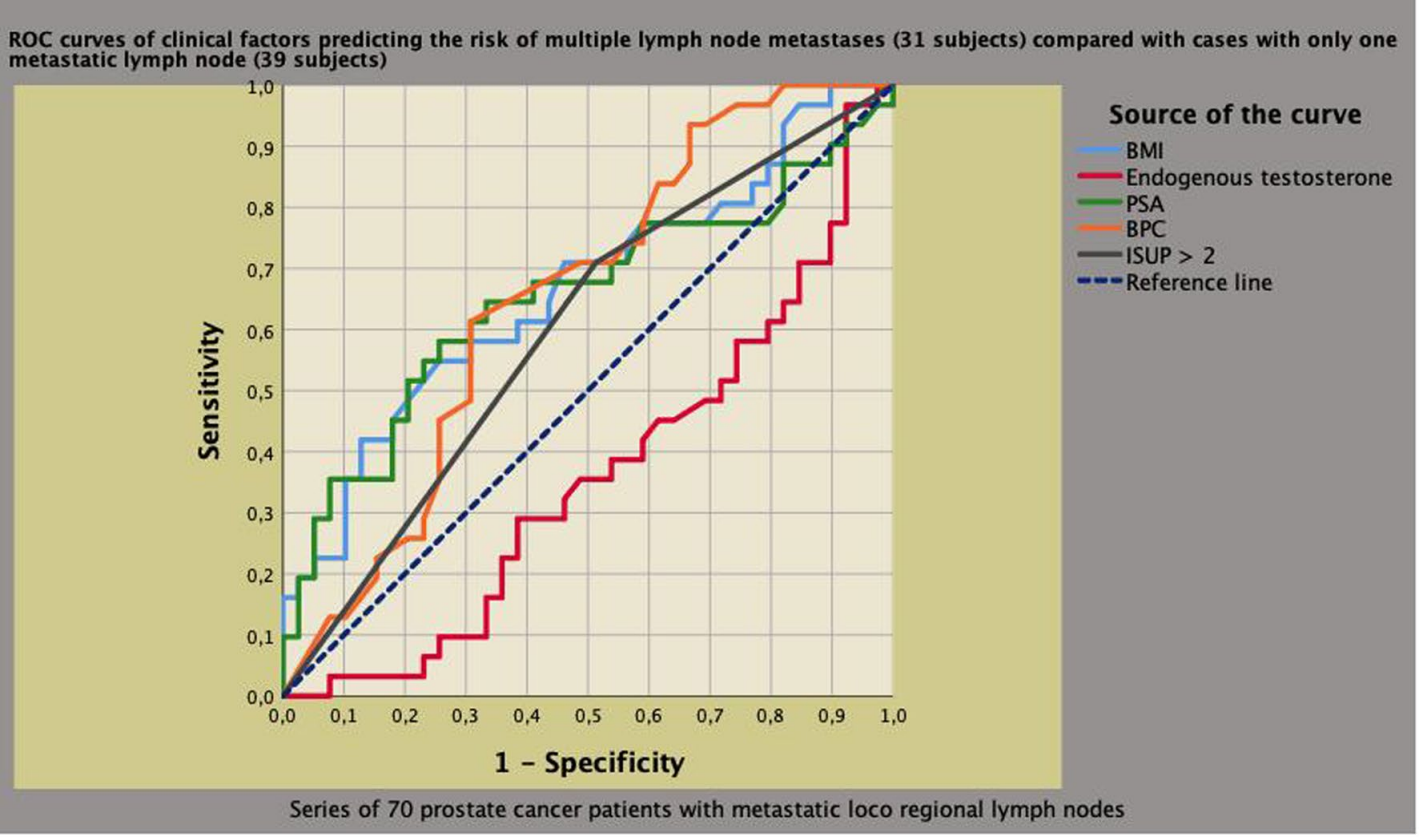
Receiver operating characteristic (ROC) curves of clinical factors predicting the risk of multiple pelvic lymph node metastases (mPLNM) compared to cases with one metastatic lymph node. Area under the curve (AUC) was 0,652 (95% CI 0.516–0.788; *p* = 0,030) for prostate specific antigen (PSA), 0,652 (95% CI 0.524–0.780; *p* = 0,030) for biopsy positive cores (BPC), 0.518 (95% CI 0.465–0.732; *p* = 0.159) for tumor grade group > 2 according to International Society of Urologic Pathology (ISUP) system, 0,665 (95% CI 0.535–0.795; *p* = 0.018) for body mass index (BMI) and 0.359 (0.229–0.489; *p* = 0,044) for endogenous testosterone (ET). So far, among metastatic patients, higher ET levels were protective for the risk of mPLNM compared to subjects with one metastatic node. See results section for further details

In metastatic patients, only ET (OR 0.994; 95% CI 0.989–1.000; *p* = 0.015) and BMI (OR 1.275; 95% CI 1.046–1.511; *p* = 0.015) remained independent predictors. ROC curves of clinical factors predicting mPLNM compared to pN0 and cases with one LNI are shown in Figs. 4 and 5, respectively. As shown, higher ET levels were protective against aggressive PCa evaluated as mPLNM in the surgical specimen.

### e) Low ET increased accuracy of multivariate models predicting the risk of mPLNM

Table 5 shows two multivariate models predicting the risk of mPLNM compared to no LNI (model I) as well as LNI with one metastatic lymph node (model II). ET, the exposure variable, is stratified by quartiles, which are referred to values above the third quartile. Considering model I, the risk of mPLNM was increased by ET levels within the first quartile (OR 4.744; 95% CI 1.073–21.234; *p* = 0.040) after adjusting for PSA, BPC and ISUP > 2; moreover, model accuracy was increased by ET up to 94.6%. Evaluating model II, the risk of mPLNM was also increased by ET levels up to the first quartile (OR 10.561; 95% CI 1.444–77.233; *p* = 0.020) after adjusting for BMI, PSA, BPC and ISUP > 2; furthermore, model accuracy improved from 64.3% (without ET) up to 74.3% (including ET). Further details are reported in Table 5.

**Table 5:** Multivariable analysis of endogenous testosterone as an exposure variable associated with the risk of multiple lymph node metastases in 617 patients with clinical prostate cancer treated by radical prostatectomy with extended pelvic lymph node dissection

Levels of the exposure variable	Median (IQR)	Metastases present in more than one lymph node versus no lymph node metastases (*)			Metastases present in more than one lymph node versus metastases in one lymph node (**)		
		Odds ratio	95% CI	*p* value	Odds ratio	95% CI	*p* value
Endogenous testosterone levels (ng/dL)	432 (340.1–535.2)						
Up to 340.1 ng/dL	279.4 (231–307.5)	4.774	1.073–21.234	0.04	10.561	1.444–77.233	0.02
Between 340.2 and 432 ng/dL	386.5 (364.5–406.3)	3.997	0.867–18.435	0.076	4.39	0.596–32.349	0.147
Between 432 and 535.2 ng/dL	479.8 (459.5–505.4)	3.997	0.880–18.162	0.073	3.932	0.568–27.218	0.165
Above 535.2 ng/dL	610.7 (558.8–683.9)	Ref			Ref		

*IQR* interquartile range; *CI* confidence interval

(*) Odds ratios calculated after adjusting for total PSA. BPC and ISUP > 2; overall model accuracy without and with endogenous testosterone being 94.8 and 94.6%, respectively

(**) Model computed after adjusting for BMI. PSA. BPC and ISUP > 2; model accuracy without and with endogenous testosterone being 64.3 and 74.3%

## Discussion

Risk of LNI above the pelvis, which is coded as stage M1a according to the actual TNM system, is higher for mPLNM than for cases with low metastatic load. Indeed, disease staged as locally advanced with mPLNM indicates aggressive biology for involving multiple lymphatic pathway networks that increase the risk of invading retroperitoneal lymph nodes, which indicate that the disease has become systemic. In our study, the median (IQR) of removed was 24 (18–32), which is far away from standard assessed limits for defining an ePLND appropriate for anatomical staging of pelvic lymph nodes; as such, our results are supported by high quality ePLND. Metastases occurred at loco-regional pelvic lymph nodes in 11.3% of cases, of whom 5% were high load for including more than one metastatic node; so far, when ePLND was performed, the rate of metastatic patients having mPLNM was 44.3%, which means that almost one out of two patients were likely to have more than one metastatic node at the pathology report. So far, ePLND increased not only clinical under-staging but also level of LNI for stratifying patients into low and high metastatic load, which is an important criterion for coding the disease as aggressive or not. We showed that ET was a further independent clinical parameter to consider beyond well-known standard predictors. The results of our study have shown that low ET levels not only are associated with the risk of mPLNM but also increased accuracy of the multivariate model; as such, ET was able to discriminate levels of pelvic metastatic load in clinical PCa staged anatomically with ePLND. Therefore, the results of our study represent a novelty of the academic literature dealing with this specific topic.

Development and progression of PCa is a multistep-process and involves several factors including genetics, lifestyle, diet and ET levels as well; however, the association between ET levels and PCa biology is a controversial issue, which still remains unsettled since controlled studies are missing; furthermore, ET levels should be measured on a chronic basis [19–21]. The association of ET with the risk of aggressive PCa biology has been reported as direct, inverse or even null; furthermore, associations with PCa have been investigated with grade, stage or cancer involving surgical margins. Associations with pN stage have also been explored with findings showing that low ET levels indicated aggressive disease for locally advanced cancers. In 1985, a British study investigating on 98 non-metastatic advanced PCa patients treated with endocrine therapy and showed that basal ET levels above the median as well as lower Gleason grade indicated better prognosis; on multivariate analysis, low ET levels and high Gleason score still associated with a poor prognosis; however, although the study included not-metastatic locally advanced tumor stage, it did not staged pelvic lymph nodes; as such, we suppose that part of the non-metastatic population with extra-prostatic extension had also locally advanced disease for LNI at pelvic nodes; so far, ET measurements indicated feasibility of selecting a poor prognostic group of patients [22]. In 2003, a North American study investigated on 879 PCa patients treated with RP showing that lower pretreatment ET associated with non-organ confined disease in the surgical specimen; furthermore, although ET was an independent predictor of extra-prostatic disease, it did predict biochemical recurrence; however, the trial was limited for lymph node dissection, which was not extended and did not reported the number of dissected and positive nodes; furthermore, associations with LNI were not investigated for the low incidence of events (only 15 cases out of 879 patients); finally, it did not control for ET circadian rhythm and BMI [7]. In 2005, another North American trial explored the relationship between pre-operative testosterone and pathological stage and progression in PCa patients treated with RP; the study showed that lower testosterone levels correlated with adverse pathological stage including extra-prostatic extension, seminal vesicle invasion and/or nodal metastasis as did standard clinical factors including PSA, clinical stage and biopsy grade; however, no relationships were detected between testosterone and biochemical recurrence after adjusting for clinical covariates; furthermore, the study was limited for being retrospective, for the cohort, which included 326 patients, for methods, since LNI was associated with advanced pathological stage; finally, lymph dissection was not extended and data on lymph nodes status (number of dissected and positive nodes) was not reported [23]. In 2005, a Japan study also investigated on pre-operative testosterone levels in 82 patients with clinically localized PCa treated with RP; the researchers detected significantly lower testosterone levels in non-organ confined disease including extra-prostatic extension (pT3 disease) with LNI compared to patients with organ confined PCa; furthermore, investigators found out that ET was an independent predictor of extra-prostatic disease associated with LNI, pathology Gleason score and PSA; however, ET did not have any impact on biochemical recurrence; moreover, the study was limited mainly for the cohort including only 82 cases, for the limits related to anatomical staging as well as for including only four patients having pN1 disease [24]. In 2011, a North European study while investigating on pre-operative testosterone levels also showed that significantly lower testosterone levels were detected in patients with LNI when compared to cases without metastases; however, the study compared patients having at the same time high grade tumors and lymph node metastases, was retrospective with the cohort including only 217 cases and did report the number of dissected and positive nodes; moreover, LNI was detected in only 19 patients [25]. In 2014, a German study investigating on ET levels in 510 men with clinically localized PCa treated with surgery find out that obesity and not ET associated with pN1 disease; however, the study had several limitations for staging (40 patients had pNx disease), for PLND not being extended, for not reporting number of dissected and positive nodes; furthermore, the association of obesity with pN1 disease was weak or not significant at all when ET was evaluated as a continuous parameter in the multivariable model [26]. So far, several studies have focused on the topic associating pre-operative ET measurements with advanced pathological stage including confined and not confined disease with the latter including extracapsular extension, seminal vesicle invasion, involvement of surgical margins and metastases at dissected pelvic lymph nodes. Compared to the above mentioned trials, our study has several distinguishing features. The quality of anatomical staging of pelvic lymph nodes was adequate for extension of the nodal field, for reporting number of dissected and metastatic nodes thus allowing to assess locally advanced disease, which was then stratified according to the metastatic load. Our study is the first showing that ET is an independent factor associating with the risk of locally advanced disease with high metastatic load at loco-regional pelvic lymph nodes. Our results also show for the first time that as ET levels decline patients have an increased likelihood of mPLNM; furthermore, low ET levels increased accuracy of multivariate models for predicting locally advanced PCa, so far, ET is a further clinical parameter to consider for future nomograms and/or neural networks investigating on this subject.

The association of locally advanced PCa with high metastatic load with low ET levels and/or increased BMI is an interesting but unsettled issue. As such, several mechanisms may be considered. Lower ET levels may be related to a general chronic disease state. PCa itself may inhibit ET levels for producing a substance called inhibin that induces a negative feedback on the hypothalamic pituitary axis. Actually, the saturation model has been proposed to explain PCa growth [27]. According to this theory, prostate growth is limited by ET levels at or below the near castrate range and maximal binding to androgen receptor (AR) occurs at these levels. As such, ET levels around or below castrate levels will induce changes in PCa, which becomes more undifferentiated and aggressive. In our opinion, mechanisms may be more complex when considering PCa developing and progressing in the human body as a system [28]. The increasing incidence of PCa with aging, which relates to decreasing levels of ET, suggests an association between the events. Low ET levels may impact on prostate microenvironment with drawbacks on induction, growth and progression to undifferentiation of PCa. The cascade of these events may be even more pivotal in obese or BMI progressing patients in whom there is an inverse correlation with ET. Prostate environment is even more stressed in overweight and obese patients for increased serum levels of inflammatory and growth factors. All these events will destabilize the saturation model and let the tumor progress to undifferentiation and locally advanced stage with high metastatic load. In smaller cohorts of patients, we have shown that obesity associated with the risk of mPLNM when anatomical staging was performed through ePLND [29]. Furthermore, we also demonstrated that overweight and obese patients were at increased risk of harboring mPLNM and lower ET levels when compared to normal weight cases [30]. The results of the present study support all these theories and for the first time show that, in large cohort of patients, as ET levels increase and BMI measurements decrease, patients have a decreased likelihood of mPLNM. So far, the findings of our study support the theory that development of PCa might be related to complex interactions, which occur along male lifetime, as a consequence of diet, increasing BMI and decreasing ET levels.

Our study has several limits. First, it is retrospective and, as such, suffers of limitations of such kind of investigations. Second, we performed ePLND in low-risk patients; however, ePLND in low risk and unfavorable intermediate risk patients is recommended by NCCN when the risk of at least 2%; furthermore, there is a set of low-risk patients who are at increased risk of tumor upgrading in the surgical specimen and, as such, ePLND is indicated for anatomical staging of pelvic lymph nodes [2, 4]. Third, the diameter of the largest metastasis and extra nodal extension of cancer was not assessed; however, number of removed and metastatic nodes are by far the most important factor for evaluating the quality of an ePLND and, as such, the anatomical staging of pelvic lymph nodes. Fourth, operations were performed by several surgeons who, however, were experienced. Fifth, ET was measured only once and not on a chronically pattern, as recommended. Although our study has limitations, it also shows strengths. First, data were collected prospectively, although being evaluated retrospectively. Second, ET measurements were all performed with our laboratory and at the same time in the morning thus avoiding biases related to diurnal testosterone variations as well as to lab methods. Third, anatomical staging of pelvic lymph nodes was appropriate for being coded as extended.

## Conclusions

In patients with clinical PCa treated with RP and ePLND, low ET levels independently associated with the risk of locally advanced disease with high load of LNI. As ET decreased patients had an increased likelihood of mPLNM and as such higher levels were protective against of aggressive disease. The influence of locally advanced PCa with high metastatic load on ET levels needs to be investigated by large multicenter prospective trials.

## References

[1] Ferlay J, Soerjomataram I, Dikshit R (2015). Cancer incidence and mortality worldwide: sources, methods and major patterns in GLOBOCAN 2012. Int J Cancer.

[2] Mottet N, Bellmunt J, Bolla M (2017). EAU-ESTRO-SIOG guidelines on prostate cancer. Part 1: screening, diagnosis, and local treatment with curative intent. Eur Urol.

[3] Fossati N, Willemse PM, Van den Broeck T (2017). The benefits and harms of different extents of lymph node dissection during radical prostatectomy for prostate cancer: a systematic review. Eur Urol.

[4] Mohler JL, Antonarakis ES, Armstrong AJ (2019). Prostate cancer, version 2.2019, NCCN clinical practice guidelines in oncology. J Natl Compr Cancer Netw.

[5] Porcaro AB, Tafuri A, Sebben M (2019). Positive association between basal total testosterone circulating levels and tumor grade groups at the time of diagnosis of prostate cancer. Urol Int.

[6] Tafuri A, Sebben M, Shakir A (2020). Endogenous testosterone mirrors prostate cancer aggressiveness: correlation between basal testosterone serum levels and prostate cancer European Urology Association clinical risk classes in a large cohort of Caucasian patients. Int Urol Nephrol.

[7] Massengill JC, Sun L, Moul JW (2003). Pretreatment total testosterone level predicts pathological stage in patients with localized prostate cancer treated with radical prostatectomy. J Urol.

[8] Dai B, Qu Y, Kong Y (2012). Low pretreatment serum total testosterone is associated with a high incidence of Gleason score 8–10 disease in prostatectomy specimens: data from ethnic Chinese patients with localized prostate cancer. BJU Int.

[9] Ferro M, Lucarelli G, Bruzzese D (2017). Low serum total testosterone level as a predictor of upstaging and upgrading in low-risk prostate cancer patients meeting the inclusion criteria for active surveillance. Oncotarget.

[10] Cabral PH, Iwamoto MW, Fanni VS (2013). Study of testosterone as a predictor of tumor aggressiveness in patients with prostate cancer. Int Braz J Urol.

[11] Briganti A, Larcher A, Abdollah F (2012). Updated nomogram predicting lymph node invasion in patients with prostate cancer undergoing extended pelvic lymph node dissection: the essential importance of percentage of positive cores. Eur Urol.

[12] Porcaro AB, Cavicchioli F, Mattevi D (2017). Clinical factors of disease reclassification or progression in a contemporary cohort of prostate cancer patients elected to active surveillance. Urol Int.

[13] Porcaro AB, Siracusano S, De Luyk N (2016). Low-risk prostate cancer and tumor upgrading to higher patterns in the surgical specimen. Analysis of clinical factors predicting tumor upgrading to higher gleason patterns in a contemporary series of patients who have been evaluated according to the modified gleason score grading system. Urol Int.

[14] Porcaro AB, Siracusano S, de Luyk N (2018). Clinical factors stratifying the risk of tumor upgrading to high-grade disease in low-risk prostate cancer. Tumori.

[15] Porcaro AB, Cacciamani GE, Sebben M (2019). Lymph nodes invasion of marcille's fossa associates with high metastatic load in prostate cancer patients undergoing extended pelvic lymph node dissection the role of marcillectomy. Urol Int.

[16] Cacciamani GE, Porcaro AB, Sebben M (2019). Extended pelvic lymphadenectomy for prostate cancer: should the Cloquet's nodes dissection be considered only an option?. Minerva Urol Nefrol.

[17] Dripps RD, Lamont A, Eckenhoff JE (1961). The role of anesthesia in surgical mortality. JAMA.

[18] Dindo D, Demartines N, Clavien P-A (2004). Classification of surgical complications: a new proposal with evaluation in a cohort of 6336 patients and results of a survey. Ann Surg.

[19] Fujita K, Nonomura N (2018) Role of androgen receptor in prostate cancer: a review 10.5534/wjmh.180040 PMC6704300 30209899

[20] Paris PL, Hofer MD, Albo G (2006). Genomic profiling of hormone-naive lymph node metastases in patients with prostate cancer. Neoplasia.

[21] Shoag J, Barbieri CE (2016). Clinical variability and molecular heterogeneity in prostate cancer. Asian J Androl.

[22] Wilson D, Harper M, Richards G (1985). A prognostic index for the clinical management of patients with advanced prostatic cancer: a British Prostate Study Group investigation. Prostate.

[23] Isom-Batz G, Bianco FJ, Kattan MW (2005). Testosterone as a predictor of pathological stage in clinically localized prostate cancer. J Urol.

[24] Imamoto T, Suzuki H, Fukasawa S (2005). Pretreatment serum testosterone level as a predictive factor of pathological stage in localized prostate cancer patients treated with radical prostatectomy. Eur Urol.

[25] Kratzik C, Womastek I, Bieglmayer C (2011). Lower serum total testosterone is associated with lymph node metastases in a radical prostatectomy cohort study. Anticancer Res.

[26] Jentzmik F, Schnoeller TJ, Cronauer MV (2014). Corpulence is the crucial factor: association of testosterone and/or obesity with prostate cancer stage. Int J Urol.

[27] Morgentaler A, Traish AM (2009). Shifting the paradigm of testosterone and prostate cancer: the saturation model and the limits of androgen-dependent growth. Eur Urol.

[28] Tafuri A, Porcaro AB, Shakir A (2021). Serum testosterone and obesity in prostate cancer biology: a call for health promotion in the ageing male. Aging Clin Exp Res.

[29] Tafuri A, Amigoni N, Rizzetto R (2020). Obesity strongly predicts clinically undetected multiple lymph node metastases in intermediate-and high-risk prostate cancer patients who underwent robot assisted radical prostatectomy and extended lymph node dissection. Int Urol Nephrol.

[30] Porcaro AB, Tafuri A, Sebben M (2020). High body mass index predicts multiple prostate cancer lymph node metastases after radical prostatectomy and extended pelvic lymph node dissection. Asian J Androl.

